# Cardiac magnetic resonance in tropical endomyocardial fibrosis

**DOI:** 10.1186/1532-429X-13-S1-P270

**Published:** 2011-02-02

**Authors:** Gurpreet S Gulati, Ajay K Sharma, Swati Paliwal, Sandeep Seth, Sivasubramanian Ramakrishnan, Priya Jagia, Sanjiv Sharma

**Affiliations:** 1 All India Institute of Medical Sciences, Ansari Nagar, New Delhi, India

## Introduction

Endomyocardial fibrosis (EMF) is a common cause of primary restrictive cardiomyopathy (RCM) in the tropics. Available studies on the role of CMR in diagnostic evaluation of EMF are confined to case reports. We describe the spectrum of CMR features in a group of patients with this disease.

## Purpose

To elucidate the role of CMR in the imaging evaluation of EMF and to devise diagnostic criteria for the disease.

## Methods

Over a period of 5 years, 100 cases of suspected RCM were referred for CMR. All patients underwent 1.5 T MRI (Magnetom Avanto, Siemens, Germany) with standard cardiomyopathy protocol: fast spin echo T1 and T2 (fat suppressed) weighted, steady state free precession and delayed enhancement (DE) images along the axial, 4-chamber, vertical long axis and short axis planes. Criteria for diagnosis of RCM included normal sized ventricles, normal/reduced systolic function, uni-/bi-atrial enlargement, normal pericardium and absent septal bounce. Cases diagnosed as EMF on CMR were included in this retrospective study. Etiology confirmation beyond CMR was not considered necessary for this diagnosis.

## Results

**Figure 1:**
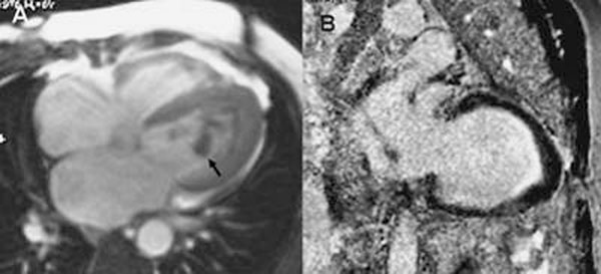
SSFP 4-chamber (A) and Delayed enhanced Phase sensitive inversion recovery image (B). There is apical obliteration of the left ventricle with thrombus (Arrow). Subendocardial enhancement is seen at the apex in B.

Of the 64 patients with RCM on CMR, EMF was diagnosed in 20 patients (31%) [12 males; age 39[[Unsupported Character - Codename &shy;]]²18 years]. Ten patients had right ventricular (RV) EMF, 3 had left ventricular (LV) EMF, while 7 had bi-ventricular EMF. Oedema indicating ongoing inflammation was seen in 4 (20%) cases. Apical thrombus was seen in 8 (40%) cases and was present in 35% cases of RV and 20% cases of LV involvement. Apical subendocardial DE was always present in the involved ventricle. The RV apex was obliterated in 100% of patients with RV involvement, while LV apex was similarly obliterated in 66 % cases with LV disease. Mild-moderate pericardial effusion was observed in 8 patients. On the basis of CMR findings, the disease was classified as early necrotic phase in 1, thrombotic necrotic in 4 and late fibrotic phase in 13 and of different stages between ventricles in 2 cases. Figure 1.

## Conclusions

EMF was the commonest cause of RCM in our series. Major diagnostic criteria of EMF on CMR include subendocardial DE and apical obliteration. Oedema and thrombus are variable findings, depending on disease severity.

